# Impact of Childhood Trauma on Insight and Medication Adherence in Patients with Schizophrenia and Schizoaffective Disorder

**DOI:** 10.1192/j.eurpsy.2026.12184

**Published:** 2026-07-31

**Authors:** E. Maaloul, R. Chakchouk, S. Ellouze, F. Sahnoun, N. Halouani, M. Turki, J. Aloulou

**Affiliations:** Psychiatry « B » Department, CHU Hedi CHaker Sfax, Sfax, Tunisia

## Abstract

**Introduction:**

Insight and medication adherence are key determinants of clinical outcome in schizophrenia spectrum disorders. Emerging evidence suggests that early adverse experiences may disrupt these processes by altering emotional processing and illness awareness.

**Objectives:**

This study intends to examine the associations between childhood trauma, insight, and medication adherence among outpatients with schizophrenia and schizoaffective disorder.

**Methods:**

A cross-sectional study was conducted on 31 outpatients diagnosed with schizophrenia or schizoaffective disorder. Childhood trauma was evaluated using the Childhood Trauma Questionnaire–Short Form (CTQ-SF), insight was measured with the Birchwood Insight Scale (BIS), and medication adherence was assessed using the Morisky Medication Adherence Scale (MMAS-4).

**Results:**

In our study, 58.1% of patients demonstrated good insight, while 41.9% exhibited poor insight. Regarding medication adherence, 22.6% of participants showed high adherence, whereas 77.4% had low to moderate adherence. Insight and medication adherence were significantly associated (p = 0.013). Higher levels of childhood trauma were observed among patients with low to moderate medication adherence (p =0.029).

**Conclusions:**

These findings emphasize the intricate interplay between trauma history, insight, and treatment engagement in schizophrenia and schizoaffective disorder. Childhood trauma appears to hinder treatment engagement, mainly by affecting medication adherence and illness awareness. Integrating trauma-informed psychoeducation and adherence-enhancing strategies into routine psychiatric care may therefore be essential to improve clinical outcomes in this vulnerable population.

**Disclosure of Interest:**

None Declared

